# Editorial: Updates in ocular therapeutics and surgery

**DOI:** 10.3389/fmed.2023.1144218

**Published:** 2023-02-14

**Authors:** Georgios D. Panos, Paris Tranos, Horace Massa

**Affiliations:** ^1^Department of Ophthalmology, Queen's Medical Centre, Nottingham University Hospitals, Nottingham, United Kingdom; ^2^Department of Retina and Uveitis, Ophthalmica Eye Hospital, Thessaloniki, Greece; ^3^Department of Ophthalmology, Geneva University Hospitals, Geneva, Switzerland

**Keywords:** cornea, retina, strabismus, ocular surface, cataract surgery, intraocular lenses, glaucoma

Ophthalmology is a field of medicine that has grown rapidly during the last few decades. Several new and promising treatments have become available for eye conditions, including age-related macular degeneration, glaucoma, retinal vein occlusions, diabetic macular oedema, genetic disorders, uveitis, and inflammations. On the other hand, recent technological developments and advanced surgical techniques have drastically changed the daily practice of eye surgeons, with new surgical options resulting in safer, faster, and more precise surgery.

This Research Topic summarized modern therapeutic and surgical approaches to ocular diseases, adding a valuable contribution to our existing knowledge, describing the current state-of-the-art, and providing directions for future research.

## Cornea and ocular surface

A systematic review and meta-analysis conducted by Sun et al. demonstrated that in patients who have already undergone corneal refractive surgery, presbyopia-correcting intraocular lenses (IOLs) deliver satisfactory outcomes in terms of efficacy, safety, and predictability, but have a higher risk of photopic side effects such halos and glare. Wy et al. compared the clinical outcomes of maximum tolerated medical therapy in patients with penetrating keratoplasty with those of Ahmed glaucoma valve implantation and found no significant differences in the visual acuity, corneal thickness and in survival time. Zhang et al. compared visual outcomes and corneal optical quality after small incision lenticule extraction (SMILE), wavefront-optimized (WFO) FS-LASIK, and topography-guided customized ablation treatment (TCAT) FS-LASIK for myopia and found that all techniques provided similar visual results with the TCAT FS-LASIK to be superior in terms of corneal optical quality. The Management of Vernal Keratoconjunctivitis in Asia (MOVIA) Expert Working Group completed a consensus program to evaluate, review, and develop best-practice recommendations for the assessment, diagnosis, and management of Vernal Keratoconjunctivitis in Asia which was published in this Research Topic (Mehta et al.).

## Glaucoma

A meta-analysis conducted by Chen X. et al. showed that XEN gel stent was effective and safe for primary and secondary open angle glaucoma with an overall complete success rate varying between 21.0 and 70.8% after 2 years and an incidence of sight-threatening complications below 1%. In a prospective case series, Chen M. et al. assessed the safety and efficiency of carbon dioxide (CO_2_) laser-assisted sclerectomy surgery (CLASS) in Chinese patients with primary open-angle glaucoma and found that CLASS was a safe and effective approach, while Xiang et al. demonstrated that that pars plana Ahmed valve implantation can be safely performed for managing vitrectomised eyes with refractory glaucoma.

## Oculoplastics and strabismus

In a prospective randomized control trial by Irawati et al., the modified tarsoraphy technique was found to be more efficient than the gold weight implant as a surgical treatment for paralytic lagophthalmos in patients with leprosy. Chi and Lai compared the endoscopic dacryocystorhinostomy with short-term, pushed-type bicanalicular intubation vs. the pulled-type monocanalicular intubation in patients with primary acquired nasolacrimal duct obstruction and found both techniques comparable in terms of surgical outcomes. Supramaximal horizontal rectus recession–resection surgery was found to be an effective treatment method for complete abduction deficiency in a retrospective study by Wang et al., while Lee et al. proposed a new simple marking system for accurate intraoperative monitoring and adjustment of cyclotorsion strabismus surgery.

## Retina

Zhou et al. found that age, disease duration, baseline central macular thickness and visual acuity are predictive factors for the visual outcomes at 6-month of anti-VEGF treatment for macular oedema secondary to retinal vein occlusion and that a further metric that can be used to forecast improved long-term outcomes is the “2-week CMT decrease rate >37%” after the initial injection. Anti-VEGF agents as the primary treatment provide potential advantages over laser therapy for eyes with zone I type 1 ROP and A-ROP, according to a large retrospective study from China by Linghu et al.. Laser photocoagulation and anti-VEGF agent therapy were equally effective for treating eyes with zone II type 1 ROP, but the rate of reactivation with laser therapy was much lower than that with anti-VEGF agents. Finally, Lumi et al. described a new technique for macular pucker peeling without forceps by using a 25-gauge vitrectomy probe.

## Cataract surgery and intraocular lenses implantation

Ye et al. described a modified technique for the scleral fixation of a secondary foldable three-piece intraocular lens, and reported successful outcomes in 10 eyes. The long-term endothelial cell count was better in low-energy femtosecond laser-assisted cataract surgery (FLACS) while the rest of the intraoperative and post-operative outcomes were comparable between these two procedures, according to Liu et al. randomized clinical trial. In a retrospective comparative study, Chen Z.-X. et al. demonstrated that supra-capsular and scleral-fixated intraocular lens (IOL) implantation was comparable to modified capsular tension ring and in-the-bag intraocular lens implantation in patients with microspherophakia. Finally, in an experimental study, Xie et al. identified primary calcification and vacuaoles as the main causes of US-860 UV and L-312 IOL opacification, respectively.

We hope that you will enjoy this Research Topic as much as we enjoyed editing it.

## Author contributions

All authors listed have made a substantial, direct, and intellectual contribution to the work and approved it for publication.

